# Design, Intervention Fidelity, and Behavioral Outcomes of a School-Based Water, Sanitation, and Hygiene Cluster-Randomized Trial in Laos

**DOI:** 10.3390/ijerph15040570

**Published:** 2018-03-22

**Authors:** Anna N. Chard, Matthew C. Freeman

**Affiliations:** Department of Environmental Health, Rollins School of Public Health, Emory University, Atlanta, GA 30322, USA; achard@emory.edu

**Keywords:** water, sanitation, hygiene, WASH, primary schools, handwashing, toilet use, behavior change, intervention fidelity

## Abstract

Evidence of the impact of water, sanitation, and hygiene (WASH) in schools (WinS) interventions on pupil absence and health is mixed. Few WinS evaluations rigorously report on output and outcome measures that allow for comparisons of effectiveness between interventions to be made, or for an understanding of why programs succeed. The Water, Sanitation, and Hygiene for Health and Education in Laotian Primary Schools (WASH HELPS) study was a randomized controlled trial designed to measure the impact of the United Nations Children’s Fund (UNICEF) Laos WinS project on child health and education. We also measured the sustainability of intervention outputs and outcomes, and analyzed the effectiveness of group hygiene activities on behavior change and habit formation. Here, we present the design and intermediate results from this study. We found the WinS project improved the WASH environment in intervention schools; 87.8% of schools received the intervention per design. School-level adherence to outputs was lower; on average, schools met 61.4% of adherence-related criteria. The WinS project produced positive changes in pupils’ school WASH behaviors, specifically increasing toilet use and daily group handwashing. Daily group hygiene activities are effective strategies to improve school WASH behaviors, but a complementary strategy needs to be concurrently promoted for effective and sustained individual handwashing practice at critical times.

## 1. Introduction

Access to water, sanitation, and hygiene (WASH) facilities and behavior change education in schools are critical for a strong learning environment, and contribute to inclusion, dignity, and equity [1]. WASH in schools (WinS) programs also support feeding programs and preventive chemotherapy to reduce reinfection with soil-transmitted helminths and trachoma [2]. As such, WinS programs are increasingly incorporated in political and development agendas as a modality to improve children’s health and boost educational attendance and achievement [3,4,5]. However, evaluations assessing the health and educational impacts of WinS have found mixed results. In Kenya, a hygiene and sanitation intervention reduced absences for girls by 58%, but not for boys [6], and had an impact on some soil-transmitted helminths [7], but not on diarrhea [8]. The arm that included water found reductions in diarrhea among both school children and their younger siblings [8,9] as well as increased enrollment and gender parity [10]. A matched-control trial of a comprehensive WinS intervention in Mali found no impact on reduced absence, but did show a reduction in self-reported diarrhea and respiratory infection [11]. In China, a comprehensive hygiene campaign where soap and peer monitoring was provided resulted in a lowered number of absences for children in the high intensity hygiene study arm, but no reduction was reported among children in the standard behavior change arm, and there was no reduction in illness among either arm [12].

There are many potential reasons for these mixed results, including environmental conditions, disease transmission dynamics, and background coverage rates [13]. WinS improvements may simply be insufficient to overcome other drivers of absence and illness, though intervention effectiveness inherently also plays a crucial role, as has been noted in several recent large-scale WASH studies [14]. Many impact evaluations report on an intervention as it would have been delivered at scale (i.e., an effectiveness study), yet few report on rigorous output and outcome measures that allow for comparison of effectiveness between interventions [15], or to understand why programs succeeded and in what context. In the Kenya and Mali WinS studies discussed above, intervention schools with higher intervention fidelity had better outcomes [16,17].

Poor sanitation and hygiene in Lao People’s Democratic Republic (Lao PDR) account for three million disease episodes and 6000 premature deaths each year [18]. In 2015, 80% percent of Laotians had access to an improved water source, while 73% of the total population had access to improved sanitation, with estimates lower in rural areas (73% and 60%, respectively) [19]. Water and sanitation access in primary schools is even worse, with functioning water and sanitation facilities available in between 29.4% and 38.9% of centers [20,21]. In the 2005 National Education Sector Development Plan, the Government of Lao PDR (GoL) set a target for improved water and sanitation access in 50% of schools by 2015. In 2013, the United Nations Children’s Fund (UNICEF) and the GoL began the Laos Basic Education, Water, Sanitation and Hygiene Programme, a four-year WinS improvement project in Lao PDR. The objective of the program was to increase school attendance through the delivery of WASH facilities to 492 schools in 13 provinces across the country, promote health and hygiene behaviors in 100 primary schools in Saravane Province, and provide improved and sustainable water access to more than 80 school-hosting villages (villages whose school received WinS programming).

The Water, Sanitation, and Hygiene for Health and Education in Laotian Primary Schools (WASH HELPS) study employed a cluster-randomized control trial with longitudinal data collection to quantify the impact of UNICEF’s WinS project on pupil learning and health in Lao PDR. Here we assessed the project’s intervention fidelity—defined as how the intervention was delivered per the stated objectives, as well as downstream school-level adherence to the intervention by teachers and students [22,23]. This paper also serves to describe the study design for our forthcoming paper assessing impact of the intervention.

## 2. Materials and Methods

### 2.1. Intervention

The intervention included both infrastructure (hardware) and behavior change (software) components. The hardware consisted of: (1) provision of a school water supply (borehole, protected dug well with pump, or gravity-fed system) and a water tank with connections to supply the toilet block and handwashing facilities; (2) school sanitation facilities, consisting of three toilet compartments designated for boys, girls, and disabled students; and (3) handwashing facilities, consisting of two sinks with taps connected to the water supply. The software component, called Hygiene Action led by Pupils in Schools (HAPiS), was implemented after the installation of the hardware components and consisted of: (1) clean drinking water, where each classroom received a ceramic water filter that was maintained and filled with water by teachers; (2) group handwashing with soap at critical times, in which schools were provided with three group handwashing tables and children were instructed to wash their hands with soap twice per day, guided by teachers in charge of hygiene activities; (3) toilet cleanliness, where pre-organized teams of students (boys and girls) performed light routine cleaning and maintenance of toilets; and (4) school compound maintenance, where teams of boys and girls cleaned the school compound, and garbage bins were used for light collection of waste. The approximate materials and labor cost of hardware installation (water supply, sanitation facilities, and handwashing facilities) per school, as estimated by UNICEF, was US $11,500 for schools that received a borehole or protected well with pump and US $16,000 for schools that received a gravity fed system; the approximate cost of software implementation was US $1500. These were paid for by UNICEF and do not include UNICEF staff costs.

### 2.2. Study Design

Though the parent UNICEF project was active in several provinces, this impact evaluation focused on Saravane, a province in the southern part of the country. Saravane was the only province where intervention activities had not yet occurred prior to the design of the study, which allowed for development of an experimental design. We employed a cluster randomized controlled trial (RCT) among 100 randomly selected schools (50 intervention, 50 comparison).

Due to the size and scope of the intervention, it was delivered in two phases. Group 1 schools received the intervention during the 2014–2015 school year, and included schools in the Ta Oy, Toumlane, Vapy, Lao Ngam, and Samoui Districts. Group 2 schools received the intervention during the 2015–2016 school year, and included schools in the Saravane, Lakhonepheng, and Khongsedone Districts. We collected data throughout the school year to account for temporal and seasonal variability (specifically, absenteeism, diarrhea, and respiratory illness). Data were collected over two (Group 2 schools) to three (Group 1 schools) years to track uptake and sustainability of facilities and behavior change. None of the school hosting villages participating in the impact evaluation received community-level WASH interventions or programming from UNICEF as part of the larger WinS project.

### 2.3. School Selection

Schools were randomly selected from a list of 222 eligible schools provided by UNICEF Lao PDR. Schools were eligible for inclusion if they met the following criteria: (1) they were located in Saravane Province; (2) were public primary schools; (3) not community-based construction schools; and (4) were lacking functional WASH facilities. Using a random number generator in Excel (Microsoft Corporation, Redmond, WA, USA), 100 schools were selected from this list for inclusion in the evaluation. The number of schools selected in each district was proportional to the number of eligible schools in each district. Following selection, schools were randomly assigned by the research manager to either the intervention group (50 schools) or the comparison group (50 schools) using a random number generator in Excel, and using stratified random sampling to ensure equal representation of control and intervention schools in each district. Given the need to plan for the intervention, we randomized the schools prior to baseline. Enumerators were blinded to this allocation at baseline.

### 2.4. Participant Selection

Within each school, a sample of 40 students from grades 3–5 were randomly selected from class registers by study enumerators using systematic stratified sampling to select equally among boy and girl pupils and among classes; however this was not always possible due to unequal enrollment in some schools. We interviewed students in grades 3–5 based on the ability of children at this grade level to reliably answer survey questions. This cohort of pupils was followed throughout the evaluation period. If a pupil in the cohort left the school during the evaluation period due to abandonment or transfer, that pupil was replaced the following academic year by another randomly selected pupil, maintaining equal pupil sex and class ratios as much as possible. Pupils in the fifth grade who advanced to secondary school at the end of each academic year were replaced by pupils in the third grade at the start of the following academic year. Some schools had fewer than 40 pupils in grades 3–5, in which case all students in grades 3–5 were included.

### 2.5. Power Calculation

Given a paucity of data on school absence in Lao PDR, we were not able to determine an estimate of the daily absence (primary outcome) within Lao PDR. As such, we utilized data from our evaluation of a school-based WASH program in Mali to estimate the necessary sample size [11]. We calculated the sample size of 40 pupils/school using Monte Carlo simulations of roll-call data, assuming 250 pupils per school, a daily absence rate of 5.6%, a within-school intra-class correlation (ICC, a measure of variability within versus between schools/pupils) of 0.09 and within pupil ICC of 0.36, and seven rounds of data collection.

Following collection of baseline data, we conducted a power analysis to calculate the minimum effect we were able to detect in absences (roll-call) and diarrhea (self-reported) among the study population using data from our true study population as opposed to the previous work in Mali. With 80% power, we will be able to detect a 1.9 percentage point (or 15%) change in absence and a 2.3 percentage point (or 21% change) in diarrhea. The power analysis was based on the estimated sample size for the entire study, projected from the sample size from Group 1 (4633 pupils for the roll-call/1323 pupils for the interview, 54 schools); baseline levels of absenteeism and diarrhea (12.4% and 10.8%, respectively); ICC for absenteeism (within-school ICC: 0.25, within-pupil ICC: 0.41) and diarrhea (within-school ICC: 0.17, within-pupil ICC: 0.36); and the projected number of rounds of data collection (including baseline) for each school (eight rounds for Group 1 schools and four rounds for Group 2 schools).

### 2.6. Ethics

The study was approved by Emory University’s Institutional Review Board (IRB0076404) and the Lao Ministry of Health’s National Institute of Public Health National Ethics Committee (No. 043 NIOPH/NECHR). Both Institutional Review Boards approved consent *in loco parentis* (in the place of the parent) signed by the school director. Pupils who were selected for the evaluation provided informed verbal assent. The evaluation is registered at clinicaltrials.gov (NCT02342860). The intervention was delivered to control schools in April 2017, after research activities ended.

### 2.7. Data Collection

Data were collected by a team of experienced enumerators who underwent rigorous training on research ethics, minimization of bias, and study tools and protocols. All data were collected using the Open Data Kit application [24] on Android-enabled mobile devices, except for the roll-call absence data, which were recorded on paper-based ledgers.

**Figure 1:**
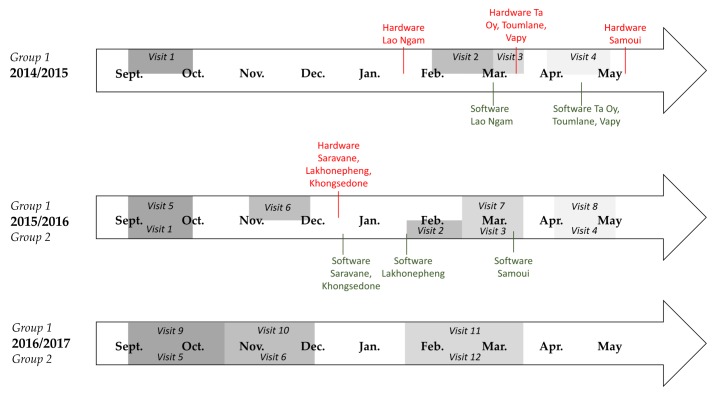
Project delivery and data collection visit timeline.

The evaluation was designed such that construction in intervention schools would occur after baseline data collection, which took place at the beginning of the school year in September/October 2014 (Group 1 schools) and September/October 2015 (Group 2 schools). Construction was expected to take approximately 8–10 weeks, with completion deadlines at the end of December (2014 for Group 1 schools and 2015 for Group 2 schools). Longitudinal surveillance of outputs, outcomes, and impacts began in February 2015 (Group 1) and 2016 (Group 2), following school exams and the January school holidays. However, given delays in construction in some schools and districts, construction was not complete in all schools by the second data collection visit, as depicted in the timeline in Figure 1.

Enumerators visited study schools every 6–8 weeks during the school year (September–May) through March 2017, for a total of 11 (Group 1) or 7 (Group 2) visits per school. On average, data were collected for 8 visits (2 years) following hardware completion in Group 1 schools, and 5 visits (1.25 years) following hardware completion in Group 2 schools. All visits were unannounced. At each visit, enumerators interviewed the school directors; interviewed up to 40 pupils in grades 3–5; observed conditions and functionality of WinS hardware; observed individual and group handwashing practices; and conducted a roll call of all students enrolled in the school.

All outputs, outcomes, and impacts, as well as their indicators and evaluation criteria were jointly developed between Emory University and UNICEF (the implementing partner) prior to the start of the study. Many, but not all, of these indicators align with the World Health Organization’s water, sanitation, and hygiene standards for schools in low-cost settings [25]. For example, the toilets were sex-separated and accessible to disabled students, but given the standard design and delivery of the toilet block and the small enrollment size of schools, we did not consider the pupil-to-latrine ratio. We measured accessibility and reliability of water points, but given that an on-site, improved water source was provided by the intervention, we did not measure water quantity. Additionally, we did not monitor water quality, which was conducted by the local water authority. We did not measure vector control or food storage/preparation, as these were beyond the scope of the intervention.

### 2.8. Baseline Measures

Baseline levels of enrollment, gender parity, school WASH access (presence of a toilet, water point in school compound, presence of handwashing facilities), school wealth, pupil demographics (age, household wealth, household presence of a toilet, use of an improved water source, and presence of a handwashing facility equipped with soap and water), and primary and secondary impacts were evaluated to ensure there were no significant differences across intervention and comparison groups and that the randomization process was successful.

Gender parity was calculated by dividing the number of boys enrolled by the number of girls enrolled in each school. School wealth was determined by the amount of money received through the School Block Grant, which is the operational budget given schools each year and is dependent on the number of pupils enrolled. Household wealth was determined through a series of questions about household construction materials (roof, floor, and walls), ownership of a mobile phone, and presence of electricity. These variables were chosen based on those used in the Demographic and Health Surveys for measures of wealth in Laos (Ministry of Health and Lao Statistics Bureau 2012). We used principal component analysis methods to derive one single wealth metric from all of the wealth assets combined [26].

### 2.9. Presence and Functionality of WinS Outputs

We collected data to measure the presence and functionality of the WinS project hardware and software outputs (Table 1). The WinS indicators and criteria defined for each output for the purpose of this evaluation go beyond the presence of infrastructure, as often defined in WASH and in evaluations, and encompass functionality and condition of the infrastructure over time, as well as adequate use (water tanks and filters must be filled; individual and group handwashing stations must be accompanied with water and soap; toilets must be kept unlocked, clean and with water available for flushing). This data was also used to assess intervention fidelity, which was defined as how well the intervention was delivered and adhered to as intended [22,23].

To measure intervention fidelity, an index score was created where one point was given for each of the 20 output criteria fulfilled. As such, for each visit, the maximum score for intervention fidelity was 20, whereas the minimum score was 0.

### 2.10. Pupil Behavioral Outcomes

We monitored five outcomes related to pupil WASH behavior change and habit formation among students: toilet use, individual handwashing, daily group handwashing, daily group toilet cleaning, daily group compound cleaning. These outcomes and their indicators are described in Table 2.

### 2.11. Health and Educational Impacts

The primary impact of interest was school absence, measured through roll-call collected by study enumerators (rather than relying on school records). Secondary impacts included pupil-reported absence, pupil-reported diarrheal incidence, pupil-reported symptoms of respiratory infection, pupil-reported absence due to illness, and soil-transmitted helminth infection. Both intention to treat and as-treated impact results from this trial will be reported in a forthcoming paper.

### 2.12. Statistical Analysis

Data were analyzed using STATA Statistical Software: Release 13 (StataCorp, College Station, TX, USA). To test for equality among intervention and comparison groups at baseline, school-level indicators were evaluated using linear (enrollment, gender parity, wealth) and logistic (school WASH access) regression. Pupil-level indicators were evaluated using linear (age, household wealth) and logistic (roll-call absence, household WASH access, reported absence, reported diarrhea, reported symptoms of respiratory infection, soil transmitted helminth infection) regression with random intercepts at the school level to account for clustering.

To measure if achievement of output and outcome indicators significantly changed among intervention schools across the evaluation period, we used logistic (binary outcomes) and linear (continuous outcomes) regression models, with random intercepts at the pupil and school levels to adjust for repeated (longitudinal) measurements, and linear splines at 7 months, 13 months, and 19 months. Programmatic adjustments were made for Group 2 schools based on lessons learned from Group 1, which led to different levels of achievement at output and outcome levels. As such, we stratify output and outcome results by implementation group. All associations were evaluated for significance at *p* < 0.05.

## 3. Results

### 3.1. Baseline

There were neither substantial nor statistically significant differences in key school- or pupil-level indicators between intervention and comparison groups at baseline, indicating that the groups were balanced after randomization allocation (Tables S1 and S2).

### 3.2. Presence and Functionality of WinS Outputs

**Figure 2:**
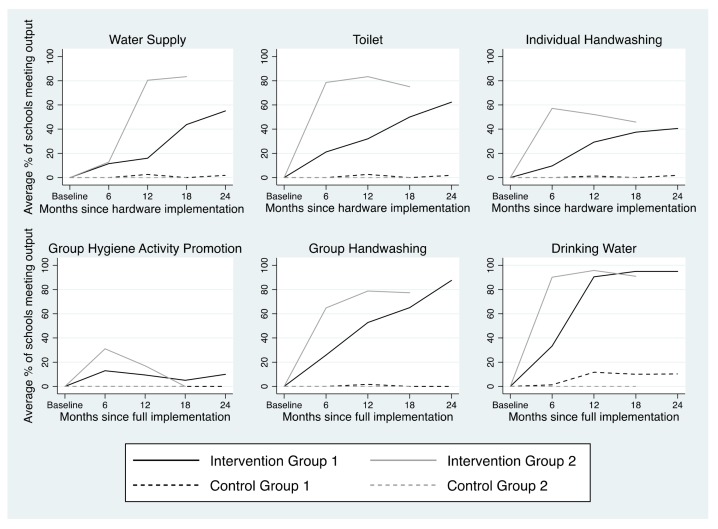
Achievement of project outputs by time since project implementation, stratified by intervention and implementation groups.

Achievement of the six project outputs across the evaluation period by intervention status and implementation group is depicted in Figure 2. Intervention schools were more likely to meet each of the indicators and evaluation criteria for the six project outputs (as described in Table 1) than were comparison schools. Generally, Group 2 intervention schools met project outputs more often than Group 1 intervention schools.

Intervention schools’ achievement of the six project outputs and their evaluation criteria throughout the evaluation period are described in Table 3. The odds of achieving project outputs and their evaluation criteria either increased or did not significantly change throughout the first six months of hardware/software implementation, with the exception of the hygiene promotion output and related criteria, the odds of which reduced throughout the first six months of software implementation in Group 1 schools. Among Group 1 schools, the odds of achieving project outputs and their criteria either continued to increase beyond six months of project implementation, or did not significantly change, indicating improved or sustained achievement, respectively. The group handwashing facility was the only criteria where odds of achievement decreased, which occurred 13–18 months after project implementation. Among Group 2 schools, achievement of most outputs and their evaluation criteria did not significantly change beyond six months. However odds of achieving some outputs/criteria increased (7–12 months: water supply output, water in tank; 13–18 months: water point did not malfunction) while others decreased (7–12 months: water point did not malfunction, water tank present, sex-separated toilets, drinking water output and associated criteria).

Of the hardware-related outputs, intervention schools were most likely to meet the toilet output (56.1% of visits after hardware implementation), followed by the handwashing output (38.6%), and the water supply output (36.4%). Of the software-related outputs, intervention schools were most likely to achieve the drinking water output (82%), followed by the group handwashing output (61%). Intervention schools were least likely to meet the promotion of group hygiene activities output (15%).

Our measure of intervention fidelity was based on achievement of the 20 criteria used to evaluate the 6 project outputs. On average, Group 1 intervention schools achieved 12.2 output criteria (95% Confidence Interval (CI) = 11.5, 12.9) after project implementation; the number of output criteria met increased by 1.2 criteria per month through the first six months following full implementation (β = 1.2, 95% CI = 1.0, 1.4), and was sustained thereafter. On average, Group 2 schools achieved 15.4 output criteria (95% CI = 14.9, 16.0); the number of output criteria met increased by 3.5 criteria for the first six months following full implementation (β = 3.5, 95% CI = 3.1, 4.0), decreased by 2.9 criteria per month between 7–12 months following full implementation (β = −2.9, 95% CI = −3.9, −1.9), and increased again 13–18 months after implementation (β = 8.7, 95% CI = 2.3, 15). Intervention schools fulfilled all six WASH outputs and their associated indicators and criteria at 2.4% of visits after project implementation; fulfillment of all 20 criteria was higher among Group 2 schools (3.6%) than Group 1 schools (1.6%, *p* < 0.01).

Quality of WinS project delivery was high; of all intervention schools (*n* = 50), 42 (87.8%) received the intervention infrastructure per design. Two (4%) did not receive a water point, three (6%) did not receive water tanks, three (6%) did not receive individual handwashing facilities, and three (6%) did not receive group handwashing facilities. School-level adherence to the outputs provided by the project (e.g., water and soap availability at handwashing facilities) was sub-optimal; of the 14 criteria related to school-level adherence, intervention schools met an average of 8.6 (Standard deviation (SD) = 3.5) criteria (61.4%) during visits following full project implementation. School-level adherence was higher among Group 2 intervention schools than Group 1 intervention schools (β: 2.3, 95% CI: 1.0, 3.7).

### 3.3. Pupil Behavioral Outcomes

**Figure 3:**
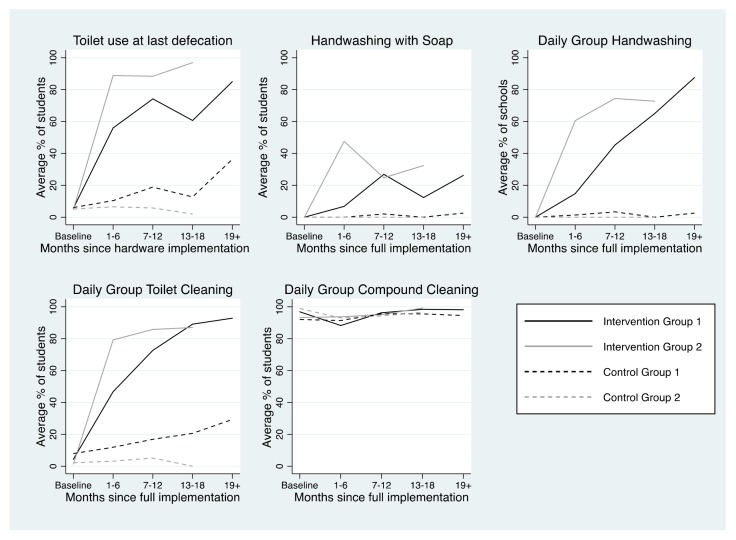
Achievement of project outcomes by time since project implementation, stratified by intervention and implementation groups.

Achievement of each of the five project outcomes by intervention status and implementation group across the evaluation period is depicted in Figure 3. After project implementation, group compound cleaning was the most commonly achieved behavioral outcome (94.8%), followed by toilet use (75.5%), group toilet cleaning (68.3%), group handwashing (48.7%), and individual handwashing with soap after toilet use (23.9%).

Trends in achievement of project outcomes among intervention schools are presented in Table 4 and described in detail below.

#### 3.3.1. Toilet Use

At baseline, only 5.9% of pupils attending intervention schools reported using a toilet at last defecation during the school day. In both implementation groups, pupil-reported toilet use at last defecation during the school day increased in the first six months following hardware implementation. In Group 1, toilet use at last defecation increased 8.5% per month between baseline and 6 months after hardware implementation (β = 8.5, 95% CI = 6.8, 10) and did not significantly change thereafter, indicating sustained behavior. In Group 2, toilet use at last defecation fluctuated across the evaluation period; it increased 20% per month from baseline to 6 months after hardware implementation (β = 20, 95% CI = 16, 24), decreased 18% per month (β = −18, 95% CI = −23, −12) from 7–12 months after hardware implementation, and increased again 34% per month (β = 34, 95% CI = 17, 50) from 13–18 months after hardware implementation.

In intervention schools, the percentage of pupils reporting toilet use at last defecation during the school day was higher among schools that met the toilet output criteria (β = 20.1, 95% CI = 14.0, 26.2). Having at least one unlocked toilet, at least one toilet with water available for flushing, and at least one clean toilet were all associated with increased prevalence of pupil-reported use of a toilet at last defecation during the school day. Having at least one gender-separated toilet compartment was not associated with reported use of a toilet at last defecation during the school day (Table S3).

#### 3.3.2. Handwashing with Soap after Toilet Use

Handwashing with soap (HWWS) after toilet use fluctuated across the evaluation period. No schools had handwashing facilities as baseline, thus HWWS was not possible. In Group 1 intervention schools, the percentage of students HWWS did not significantly change until 18+ months after software implementation, when it decreased 3.7% per month (β = −3.7, 95% CI = −7.2, −0.2). Among Group 2 intervention schools, the percentage of students HWWS increased 4.5% per month in the first six months following software implementation (β = 4.5, 95% CI = 1.9, 7.1), then decreased 7–12 months after software implementation (β = −9.8, 95% CI = −15, −4.7), and increased again 13–18 months after software implementation (β = 34, 95% CI = 1.1, 67, Table 4). The percentage of students observed to HWWS after toilet use was higher among schools that practiced group handwashing on the day of the visit (β = 31.7, 95% CI = 24.0, 39.5).

#### 3.3.3. Group Handwashing

Among Group 1 intervention schools, the odds of intervention schools conducting group handwashing did not increase until 7–12 months after software implementation (Odds Ratio (OR) = 1.8, 95% CI = 1.3, 2.4), and was sustained thereafter. Among Group 2 schools, the odds of intervention schools conducting group handwashing (GHW) increased in the first 6 months after software implementation (OR = 1.3, 95% CI = 1.0, 1.7), was sustained 7–12 months after software implementation, and slightly decreased 13–18 months after software implementation (OR = 0.6, 95% CI = 0.0, 7.3). Intervention schools were more likely to conduct GHW on the day of the visit if they had a posted schedule for GHW (OR = 4.1, 95% CI = 2.0, 8.1).

#### 3.3.4. Group Toilet Cleaning

In Group 1 intervention schools, the percentage of students reporting participating in group toilet cleaning (GTC) in the previous week increased in the first six months following software implementation, and was sustained thereafter (β = 8.5, 95% CI = 6.7, 10). In Group 2, the percentage increased in the first six months after software implementation (β = 16, 95% CI = 12, 20), declined 7–12 months after software implementation (β = −12%, 95% CI = −19, −5.6), and was sustained thereafter. Odds of pupils in intervention schools reporting participating in GTC in the previous week were higher in schools where a GTC schedule was posted (OR = 3.2, 95% CI = 2.7, 3.8). Further, there was a positive association between toilet cleanliness and GTC; toilets were more likely to be observed to be clean in intervention schools among schools where a greater percentage of students reported participating in GTC in the previous week (β = 0.4, 95% CI = 0.1, 0.6).

#### 3.3.5. Group Compound Cleaning

Student-reported participation in group compound cleaning (GCC) was high at baseline (96.9%). In Group 1 intervention schools, the percentage of students reporting participating in GCC in the previous week increased in the first six months after software implementation (β = 11, 95% CI = 9.3, 14), and was sustained thereafter. There was no significant change in the percentage of students in Group 2 schools reporting participating in GCC across the evaluation period. Odds of pupils in intervention schools reporting participating in GCC in the previous week were higher in schools where a GCC schedule was posted (OR = 2.4, 95% CI = 1.8, 3.3).

## 4. Discussion

This impact evaluation provided evidence that the UNICEF Lao PDR WinS project improved the WASH environment in intervention schools by increasing access to toilets, handwashing facilities, and safe drinking water and these improvements were sustained over two years after implementation of the project. We found that the project produced positive changes in pupils’ WASH behaviors. Specifically, the project led to increases in pupils reporting using the toilet for defecation during the school day (as opposed to open defecation), increased prevalence of pupils’ handwashing with soap following toilet use, and habitualization of daily group handwashing. Quantifying intervention fidelity is a critical component of assessing the impact of large-scale public health interventions. A priori determined output and outcome indictors agreed between government, implementation, and evaluation partners facilitated a better understanding of context specific intervention impact and provides important information to policy makers and donors.

### 4.1. Intervention Fidelity: Presence and Functionality of WinS Outputs

We found that quality of WinS project delivery was high, with 87.8% of schools receiving the intervention per stated design. School-level adherence to the outputs provided by the project was lower, but generally improved across the evaluation period. Similar results of high project delivery but low school-level adherence have been reported for school WASH projects in Mali and Kenya [16,17,27] and may be a key reason for inconsistent impact findings. WinS projects must focus on higher adherence; possibly through more appropriate technology, improving behavior change, or more accountability within the schools.

The greatest barrier to meeting the water supply and toilet outputs was water availability. Although functionality of the water point was relatively high (82% of post-hardware implementation visits), and consistent with other low-income school settings [28,29,30], schools were sometimes unable to fill the water tanks. Since the water tank supplied the handwashing facilities and the toilet compartments, water was often not available for handwashing or toilet flushing/cleaning. One reason for this was that the initial intervention design delivered to Group 1 schools consisted of a rainwater tank to supply the toilets with water. However, rainwater could not provide a consistent supply of water to fill the tank, causing pupils to have to manually fill the water tank. Thus, UNICEF revised the design, incorporating the lessons learned from the first year of intervention delivery, and detached the water tank from rain water harvesting system and connected tanks with motorized hand pumps or gravity-fed water supply systems. These results highlight the importance of routine monitoring to ensure that intervention technologies are contextually specific and appropriate. Following this adjustment, the presence of water in the water tank, in toilet compartments, and supplying the handwashing facilities improved, but was still not universal, probably because operating the pumps still required some action on part of the schools, which were not consistently performed.

Provision of soap was another adherence-related challenge; soap was observed at individual handwashing facilities during only 39.7% of post-hardware implementation visits, and the provision of soap at handwashing facilities showed little improvement as time since implementation passed. Each intervention school received one bar of soap per pupil, which was estimated to be a sufficient supply for an entire school year. Schools were expected to provide their own soap beyond this initial supply. Anecdotally, school directors reported difficulty in keeping soap by the individual handwashing facilities because of theft and of consumption by animals. Purchasing soap to supply the handwashing facilities once the initial supply ran out could have also been a financial challenge for schools or an indicator of poor buy-in from teachers and parents. Having a sufficient and consistent supply of soap is a requisite to ensure that HWWS is a habitualized practice among students. Future WinS programming could explore strategies for protecting soap from theft or animal consumption. WinS implementers should also consider additional ways to help schools maintain a consistent supply of soap that is sustainable and is not a financial burden, such as including soap making in project activities.

Lastly, few schools had schedules for daily group hygiene activities (handwashing, toilet cleaning, compound cleaning), an output that relied solely on school adherence. However, for all of the group hygiene activities, odds of the respective activity being observed (group handwashing) or reported by pupils (group toilet and compound cleaning) were significantly higher in intervention schools that had a schedule posted for the respective activity. These results suggest that posting daily group activity schedules may serve as a visual cue for school directors and students, leading to increased adherence to these activities. Given the minimal cost and time needed to make and post schedules for the daily group activities output, as well as the direct linkage to positive WASH behaviors, meeting this output could be a focus in future programming.

### 4.2. Pupil Behavioral Outcomes

The WinS project was effective in achieving behavior change on the part of the pupils. Reported toilet use for defecation during the school day increased among both intervention groups. Toilet use at last defecation during the school day increased as the number of unlocked toilets increased, a trend that has also been reported in Kenya [31]. Beyond toilets being unlocked (which is necessary for pupil use), cleanliness and water availability were the largest predictors of whether pupils reported using the toilet at last defecation during the school day. The few existing studies examining the links between toilet cleanliness and toilet use corroborate these results. In two different WinS studies in Kenya, dirty toilets were also found to be deterrents for toilet use, particularly among girl pupils [31,32]. These results suggest that promoting toilet cleanliness is an important component of WinS interventions. Interventions utilizing pour flush toilets (such as this one) should also prioritize water availability, which is necessary for flushing and maintaining clean toilet environments.

Handwashing with soap (HWWS) is a notoriously difficult behavior to improve and sustain. Three school-based studies—two in Kenya and one in Mali—have reported HWWS rates of 38%, 32–38%, and 58%, respectively [33,34,35]. In Laos, improvements in HWWS after toilet use were observed among students in intervention schools 1–6 and 13–18 months following software implementation (Group 2), but these improvements were not sustained across the evaluation period. A similar overall trend was reported in Mali, where peak handwashing was observed 7–12 months following intervention implementation, and declined thereafter [35]. Thus, although HWWS showed a positive change among pupils in intervention schools, these results point to the need to reinforce HWWS behaviors periodically throughout the school year and from one year to the next one, beyond the timeframe of any externally-supported project. Activities such as regular teacher training, administrative incentives, and appropriate follow-up, monitoring, and supervision, can be employed so that the HWWS education and promotion persists despite frequent turnover of pupils and teachers. Additionally, our results indicate what is well known in the sector: due to lack of soap, handwashing projects are unlikely to be sustained beyond the direct implementation period. While handwashing with soap is considered a cost-effective way to prevent illness, an assessment of long-term cost-effectiveness of HWWS interventions at schools may not indicate that current approaches are effective.

Daily group handwashing (GHW) was integrated into the UNICEF and German Corporation for International Development (GIZ) Three-Star Approach to WinS in 2013, however, few projects have evaluated behavioral outcomes associated with this approach. We found evidence of improved and sustained GHW behavior change across the evaluation period. Additionally, pupils attending schools where GHW was conducted on the day of the visit were more likely to practice individual HWWS after toilet use. These results point to the success of the WinS project in promoting HWWS through GHW, and suggest that GHW is an effective approach for promoting HWWS at critical times. However, more robust evaluations on the effectiveness, cost-effectiveness, and sustainability of these programmatic approaches are warranted to verify and complement the external validity of results from this evaluation.

### 4.3. Strengths and Limitations

The presence and functionality of the water point relied on report by the school director*.* We intended to include both reported and observed functionality of the water point, but due to an oversight the observation component was not included. We did observe whether the handwashing (group and individual) taps and the taps within the toilet compartments were functioning, as well as whether water was present in the water tank. Since these taps are connected to the school water supply, we were able to use handwashing and toilet functionality data to triangulate and confirm the reported water point functionality data. A second limitation was the staggered delivery of the intervention across two different school years. This could be seen as a strength of the intervention approach, as lessons learned from evaluation of the first implementation group (Group 1) were used to improve delivery to the second implementation group (Group 2). However, this did create minor limitations to the analysis; Group 2 schools often performed better than Group 1 schools in meeting output and outcome indicators. Additionally, differences in delivery also limit our ability to report on the sustainability of the intervention, as Group 1 had a full extra year of surveillance but implementation was also delayed in some districts. In order to have an accurate measure of WinS hardware and software performance and sustainability, we ideally would need to follow a single cohort of schools over the same time period. Lastly, given the quantitative design of the study, we were unable to take into account some dimensions of project delivery and adherence, specifically the dose of hygiene education received and participant responsiveness to the project [22,23]. Additionally, we were unable to explore possible socio-cultural explanations for why certain behaviors improved (e.g., toilet use), while others, such as handwashing, did not. Previous research has shown that emotional drivers and social norms can be motivators for handwashing behaviors, whereas heath or fear of disease generally are not [36,37]. WinS programming should consider these drivers prior to program design in order to ensure the Theory of Change is contextually and culturally targeted.

Despite these limitations, the design, methods, and approach of the WASH HELPS Study were robust. This is the first evaluation of a comprehensive school WASH project in Laos and one of the largest and most comprehensive evaluations to date of a school WASH project in low-income settings. Our study design—a randomized-controlled trial—is the gold standard of epidemiological evidence, and we followed schools over 2 to 3 years in order to account for inter-seasonal and inter-year variations.

## 5. Conclusions

Our results describe the success of the UNICEF Laos WinS project in improving the WASH environment in schools that were lacking WASH facilities and the effectiveness of the intervention in positively changing WASH behaviors. Similar to previous WinS impact evaluations in Mali and Kenya, we report high quality of project delivery such as provision of a functional water supply, toilets, and handwashing facilities. Conversely, there was sub-optimal school-level adherence to project outputs such as soap provision, water availability, and promoting group hygiene activities. Despite these shortcomings, most behavioral outcomes (toilet use and daily group hygiene activities) improved and/or were sustained across the evaluation period. Strategies to sustain handwashing behaviors beyond the initial 6 to 12 months of project implementation and to sustain a consistent supply of soap warrant further exploration and should be a priority for policy makers and WinS project implementers.

## Figures and Tables

**Table 1:** Water, sanitation, and hygiene (WASH) in schools (WinS) project outputs and indicators.

Output	Indicator and Criteria
**Hardware**	
Water Supply	Improved ^1^ water point on school compound ^2^ ◦ Water point functional in the previous year (director reported) ^3^ Water tank to supply toilet and handwashing stations ^2^ ◦ Water observed in tank ^3^
Toilets	At least one improved ^1^ toilet compartment ^2^ ◦ Toilet is sex separated (by designation) ^3^ ◦ Toilet is unlocked ^3^ ◦ Toilet is clean ^3^ ◦ Toilet has water available inside compartment for flushing ^3^
Handwashing facilities	At least one individual handwashing station available to pupils ^2^ ◦ Water available at individual handwashing station ^3^ ◦ Soap available at individual handwashing station ^3^
**Software**	
Promotion of daily group hygiene activities	Daily group handwashing schedule posted in at least one classroom or near toilet ^3^ Daily group compound cleaning schedule posted in at least one classroom or near toilet ^3^ Daily group toilet cleaning schedule posted in at least one classroom or near toilet ^3^
Group handwashing	Group handwashing facility available to pupils ^2^ ◦ Water available at group handwashing facility ^3^ ◦ Soap available at group handwashing facility ^3^
Water filters	At least one drinking water filter available in a classroom for pupil use ^1^ ◦ Water in filter ^2^

^1^ Defined according to Joint Monitoring Programme (JMP) standards. ^2^ Classified as quality of project delivery. ^3^ Classified as school-level adherence.

**Table 2:** WinS behavioral outcomes and indicators.

Outcome	Indicator
Toilet use	Percentage of students using toilet for defecation during school hours (pupil-reported)
Handwashing (individual)	Percentage of students washing hands with soap and water upon exiting toilet (observation)
Daily group handwashing	School conducted daily group handwashing the day of visit (observation)
Daily group toilet cleaning	Percentage of students participating in daily group toilet cleaning within the previous five school days (pupil-reported)
Daily group compound cleaning	Percentage of students participating in daily group compound cleaning within the previous five school days (pupil-reported)

**Table 3:** Per month change in odds of intervention schools achieving project output or evaluation criteria by time since project implementation.

	Group 1 Intervention Schools (*n* = 26)				Group 2 Intervention Schools (*n* = 24)		
Output or Evaluation Criteria	1–6 Months	7–12 Months	13–18 Months	19+ Months	1–6 Months	7–12 Months	13–18 Months
**Water supply output ^1^**	1.1 (0.8, 1.4)	0.9 (0.7, 1.2)	***1.6* (*1.2*, *2.1*)**	1.0 (0.8, 1.4)	1.3 (0.8, 2.0)	***2.6* (*1.4*, *4.8*)**	0.3 (0.0, 1.9)
Water point located on school grounds	***1.8* (*1.2*, *2.8*)**	1.2 (0.6, 2.2)	2.3 (0.9, 6.4)	0.5 (0.1, 1.7)	--	--	--
*Did not malfunction in previous year*	***1.4* (*1.1*, *1.6*)**	1.2 (0.9, 1.6)	0.8 (0.6, 1.1)	1.1 (0.7, 1.5)	***3.1* (*1.9*, *5.0*)**	***0.2* (*0.1*, *0.5*)**	***7.1* (*1.1*, *47*)**
Water tank	***4.4* (*1.3*, *15*)**	0.4 (0.1, 1.4)	1.7 (0.6, 4.7)	0.8 (0.2, 3.1)	***8.8* (*1.7*, *46*)**	***0.2* (*0.0*, *0.9*)**	--
*Water in tank*	1.3 (1.0, 1.6)	0.8 (0.6, 1.1)	***2.6* (*1.7*, *3.9*)**	0.9 (0.6, 1.5)	0.8 (0.5, 1.3)	***7.8* (*3.5*, *18*)**	--
**Toilet output ^1^**	1.0 (0.8, 1.3)	***1.4* (*1.1*, *1.9*)**	1.2 (0.9, 1.6)	1.1 (0.7, 1.5)	***1.6* (*1.2*, *2.0*)**	0.7 (0.5, 1.1)	1.1 (0.3, 4.7)
At least one improved toilet compartment	***4.4* (*1.9*, *10*)**	--	--	--	--	--	--
*Sex separated*	***1.4* (*1.1*, *1.8*)**	***1.5* (*1.0*, *2.1*)**	1.4 (0.8, 2.2)	0.9 (0.5, 1.9)	***7.9* (*1.7*, *37*)**	***0.1* (*0.0*, *0.9*)**	--
*Unlocked*	***1.3* (*1.0*, *1.6*)**	1.2 (0.9, 1.6)	***1.8* (*1.1*, *3.1*)**	0.8 (0.4, 1.6)	***1.8* (*1.4*, *2.5*)**	0.7 (0.4, 1.2)	1.7 (0.2, 13)
*Clean*	1.2 (1.0, 1.4)	***1.4* (*1.0*, *1.8*)**	1.3 (0.9, 1.8)	0.9 (0.6, 1.5)	***1.7* (*1.3*, *2.3*)**	0.7 (0.4, 1.2)	2.0 (0.3, 14)
*Water available inside for flushing*	***1.2* (*1.0*, *1.5*)**	1.0 (0.8, 1.3)	***1.3* (*1.0*, *1.7*)**	1.1 (0.8, 1.7)	***1.6* (*1.2*, *2.0*)**	0.7 (0.5, 1.1)	1.1 (0.3, 4.7)
**Handwashing output ^1^**	***1.4* (*1.1*, *1.8*)**	1.2 (0.9, 1.7)	1.0 (0.8, 1.4)	1.0 (0.7, 1.5)	***1.3* (*1.0*, *1.7*)**	0.7 (0.5, 1.1)	1.7 (0.5, 5.8)
At least one individual handwashing station	***4.8* (*1.5*, *16*)**	0.5 (0.2, 1.5)	1.3 (0.5, 3.6)	0.9 (0.2, 3.9)	***3.4* (*1.6*, *7.5*)**	0.4 (0.1, 1.2)	--
*Water*	***1.4* (*1.1*, *1.8*)**	1.0 (0.8, 1.4)	***1.4* (*1.0*, *1.9*)**	1.2 (0.7, 1.8)	***1.7* (*1.3*, *2.3*)**	0.7 (0.4, 1.0)	1.2 (0.3, 5.3)
*Soap*	1.3 (1.0, 1.7)	1.2 (0.9, 1.7)	1.0 (0.8, 1.3)	1.0 (0.7, 1.5)	***1.3* (*1.0*, *1.7*)**	0.7 (0.5, 1.1)	1.7 (0.5, 5.8)
**Hygiene promotion output ^2^**	***0.5* (*0.3*, *0.8*)**	***2.2* (*1.2*, *4.0*)**	0.7 (0.5, 1.0)	1.0 (0.6, 1.7)	1.0 (0.7, 1.3)	0.8 (0.4, 1.6)	--
Group compound cleaning schedule	***0.9* (*0.8*, *1.0*)**	1.0 (0.8, 1.3)	1.1 (0.9, 1.3)	1.1 (0.8, 1.6)	1.0 (0.8, 1.2)	0.9 (0.6, 1.3)	3.4 (0.3, 39)
Group toilet cleaning schedule	***0.7* (*0.6*, *0.9*)**	***1.4* (*1.1*, *2.0*)**	0.9 (0.7, 1.2)	1.2 (0.8, 1.6)	1.0 (0.8, 1.3)	0.9 (0.6, 1.4)	0.1 (0.0, 2.9)
Group handwashing schedule	***0.7* (*0.6*, *1.0*)**	1.3 (0.9, 1.9)	0.9 (0.6, 1.2)	1.0 (0.6, 1.6)	0.9 (0.7, 1.1)	1.0 (0.6, 1.8)	--
**Drinking water output ^2^**	***5.4* (*3.0*, *9.9*)**	1.2 (0.7, 2.0)	0.8 (0.5, 1.4)	1.1 (0.5, 2.4)	***3.1* (*1.9*, *5.1*)**	***0.3* (*0.1*, *0.6*)**	--
At least one drinking water filter	***8.2* (*4.8*, *14*)**	1.0 (0.5, 2.0)	--	--	***4.5* (*2.0*, *10*)**	***0.2* (*0.1*, *0.6*)**	--
*Water in filter*	***5.4* (*3.0*, *9.9*)**	1.2 (0.7, 2.0)	0.8 (0.5, 1.4)	1.1 (0.5, 2.4)	***3.1* (*1.9*, *5.1*)**	***0.3* (*0.1*, *0.6*)**	--
**Group handwashing output ^2^**	1.0 (0.8, 1.2)	***1.6* (*1.2*, *2.1*)**	0.9 (0.7, 1.2)	1.3 (0.8, 2.0)	***1.2* (*1.0*, *1.5*)**	1.2 (0.8, 1.8)	0.6 (0.0, 9.9)
Group handwashing facility	1.6 (1.2, 2.1)	***1.9* (*1.1*, *3.0*)**	***0.8* (*0.5*, *1.5*)**	--	***1.9* (*1.3*, *2.6*)**	0.8 (0.4, 1.8)	--
*Water*	***1.3* (*1.1*, *1.6*)**	1.2 (0.9, 1.5)	1.4 (1.0, 2.1)	0.9 (0.5, 1.9)	***1.4* (*1.1*, *1.8*)**	1.0 (0.6, 1.5)	0.6 (0.0, 13)
*Soap*	1.0 (0.8, 1.2)	***1.7* (*1.3*, *2.2*)**	0.9 (0.7, 1.2)	1.3 (0.8, 2.0)	1.2 (1.0, 1.5)	1.1 (0.8, 1.7)	0.6 (0.0, 11)
Number of outputs met (range 0–20) ^3^	***1.2* (*1.0*, *1.4*)**	0.1 (−0.1, 0.4)	0.2 (0.0, 0.5)	0.1 (−0.3, 0.5)	***3.5* (*3.1*, *4.0*)**	**−*2.9* (−*3.9*, −*1.9*)**	***8.7* (*2.3*, *15*)**
School-level adherence outputs met (range 0–14) ^3^	***0.7* (*0.6*, *0.9*)**	0.2 (0.0, 0.4)	0.2 (0.0, 0.4)	0.1 (−0.2, 0.4)	***1.4* (*1.0*, *1.8*)**	**−*1.0* (−*1.6*, −*0.4*)**	2.3 (−1.5, 6.1)

Bold italicization indicates significant change in odds within time strata (*p* < 0.05). -- indicates data were too sparse to calculate odds. ^1^ Analyzed by time since hardware implementation. ^2^ Analyzed by time since full implementation. ^3^ β coefficients represent the per month change in the number of outputs met within time strata.

**Table 4:** Per month change in achievement project outcomes among intervention schools by time since project implementation.

	Group 1 Schools (*n* = 26)				Group 2 Schools (*n* = 24)		
	1–6 Months	7–12 Months	13–18 Months	19+ Months	1–6 Months	7–12 Months	13–18 Months
Percentage of students reporting toilet use at last defecation during school day ^1^	***8.5* (*6.8*, *10*)**	0.1 (−2.2, 2.5)	0.3 (−1.8, 2.4)	1.8 (−1.1, 4.6)	***20* (*16*, *24*)**	**−*18* (−*23*, −*12*)**	***34* (*17*, *50*)**
Percentage of students observed handwashing with soap ^2^	1.1 (−0.1, 2.3)	0.0 (−2.5, 2.5)	−1.5 (−3.7, 0.7)	**−*3.7* (−*7.2*, −*0.2*)**	***4.5* (*1.9*, *7.1*)**	**−*9.8* (−*15*, −*4.7*)**	***34* (*1.1*, *67*)**
School observed conducting group handwashing ^2^	1.2 (0.9, 1.5)	***1.8* (*1.3*, *2.4*)**	1.0 (0.7, 1.2)	1.4 (0.9, 2.1)	***1.3* (*1.0*, *1.7*)**	1.1 (0.7, 1.6)	***0.6* (*0.0*, *7.3*)**
Percentage of students reporting participating in group toilet cleaning in previous week ^2^	***8.5* (*6.7*, *10*)**	1.8 (−1.1, 4.8)	0.7 (−2.1, 3.5)	1.4 (−2.9, 5.7)	***16* (*12*, *20*)**	**−*12* (−*19*, −*5.6*)**	39 (−0.1, 79)
Percentage of students reporting participating in group compound cleaning in previous week ^2^	***11* (*9.3*, *14*)**	−2.4 (−5.9, 1.1)	1.0 (−2.4, 4.3)	0.0 (−5.2, 5.2)	0.5 (−1.2, 2.1)	0.2 (−2.7, 3.0)	4.6 (−13, 22)

All β coefficients represent the per month change in percent of students engaging in behavior within time strata, except for group handwashing, which is a per month change in odds of school conducting group handwashing. Bold italicization indicates significant change in outcome within time interval (*p* < 0.05). ^1^ Analyzed by time since hardware implementation. ^2^ Analyzed by time since full implementation.

## Supplementary Materials

The following are available online at http://www.mdpi.com/1660-4601/15/4/570/s1, Table S1: Key school-level indicators by intervention status at baseline, Table S2: Key pupil-level indicators by intervention status at baseline, Table S3: Associations between school toilet output criteria and percentage of pupils reported toilet use for last defecation during the school day.

## References

[1] Jasper C., Le T.-T., Bartram J. (2012). Water and sanitation in schools: A systematic review of the health and educational outcomes. Int. J. Environ. Res. Public Health.

[2] Garn J.V., Mwandawiro C.S., Nikolay B., Drews-Botsch C.D., Kihara J.H., Brooker S.J., Simiyu E.W., Okoyo C., Freeman M.C. (2016). *Ascaris lumbricoides* Infection Following School-Based Deworming in Western Kenya: Assessing the Role of Pupils' School and Home Water, Sanitation, and Hygiene Exposures. Am. J. Trop. Med. Hyg..

[3] UNDP Goal 6: Clean Water and Sanitation. http://www.undp.org/content/undp/en/home/sustainable-development-goals/goal-6-clean-water-and-sanitation.html.

[4] UNICEF (2012). Raising Even More Clean Hands: Advancing Health, Learning and Equity through WASH in Schools.

[5] Freeman M.C., Ogden S., Jacobson J., Abbott D., Addiss D.G., Amnie A.G., Beckwith C., Cairncross S., Callejas R., Colford J.M. (2013). Integration of water, sanitation, and hygiene for the prevention and control of neglected tropical diseases: A rationale for inter-sectoral collaboration. PLoS Negl. Trop. Dis..

[6] Freeman M.C., Greene L.E., Dreibelbis R., Saboori S., Muga R., Brumback B., Rheingans R. (2012). Assessing the impact of a school-based water treatment, hygiene and sanitation programme on pupil absence in Nyanza Province, Kenya: a cluster-randomized trial. Trop. Med. Int. Health.

[7] Freeman M.C., Clasen T., Brooker S.J., Akoko D.O., Rheingans R. (2013). The Impact of a School-Based Hygiene, Water Quality and Sanitation Intervention on Soil-Transmitted Helminth Reinfection: A Cluster-Randomized Trial. Am. J. Trop. Med. Hyg..

[8] Freeman M.C., Clasen T., Dreibelbis R., Saboori S., Greene L.E., Brumback B., Muga R., Rheingans R. (2013). The impact of a school-based water supply and treatment, hygiene, and sanitation programme on pupil diarrhoea: A cluster-randomized trial. Epidemiol. Infect..

[9] Dreibelbis R., Greene L.E., Freeman M.C., Saboori S., Chase R.P., Rheingans R. (2013). Water, sanitation, and primary school attendance: A multi-level assessment of determinants of household-reported absence in Kenya. Int. J. Educ. Dev..

[10] Garn J.V., Greene L.E., Dreibelbis R., Saboori S., Rheingans R., Freeman M.C. (2013). A cluster-randomized trial assessing the impact of school water, sanitation, and hygiene improvements on pupil enrollment and gender parity in enrollment. J. Water Sanit. Hyg. Dev..

[11] Trinies V., Garn J., Chan H., Freeman M. (2016). The impact of a comprehensive school WASH program on absenteeism, diarrhea, and respiratory infection symptoms: A matched-control trial in Mali. Am. J. Trop. Med. Hyg..

[12] Bowen A., Ma H., Ou J., Billhimer W., Long T., Mintz E., Hoekstra M., Luby S.P. (2007). A cluster-randomized controlled trial evaluating the effect of a handwashing-promotion program in Chinese primary schools. Am. J. Trop. Med. Hyg..

[13] Fuller J.A., Eisenberg J.N. (2016). Herd protection from drinking water, sanitation, and hygiene interventions. Am. J. Trop. Med. Hyg..

[14] Clasen T., Boisson S., Routray P., Torondel B., Bell M., Cumming O., Ensink J., Freeman M., Jenkins M., Odagiri M. (2014). Effectiveness of a rural sanitation programme on diarrhoea, soil-transmitted helminth infection, and child malnutrition in Odisha, India: A cluster-randomised trial. Lancet Glob. Health.

[15] Garn J.V., Sclar G.D., Freeman M.C., Penakalapati G., Alexander K.T., Brooks P., Rehfuess E.A., Boisson S., Medlicott K.O., Clasen T.F. (2017). The impact of sanitation interventions on latrine coverage and latrine use: A systematic review and meta-analysis. Int. J. Hyg. Environ. Health.

[16] Garn J.V., Trinies V., Toubkiss J., Freeman M.C. (2017). The Role of Adherence on the Impact of a School-Based Water, Sanitation, and Hygiene Intervention in Mali. Am. J. Trop. Med. Hyg..

[17] Garn J.V., Brumback B.A., Drews-Botsch C.D., Lash T.L., Kramer M.R., Freeman M.C. (2016). Estimating the effect of school water, sanitation, and hygiene improvements on pupil health outcomes. Epidemiology.

[18] Water and Sanitation Program Findings from Hygiene and Sanitation Financing Study in Lao PDR. http://www-wds.worldbank.org/external/default/WDSContentServer/WDSP/IB/2012/10/31/000425962_20121031151510/Rendered/PDF/734140BRI0WSP00ancing0study0Lao0PDR.pdf.

[19] WHO/UNICEF Joint Monitoring Programme (2015). Lao People's Democratic Republic: Estimates on the Use of Water Sources and Sanitation Facilities (1980–2015).

[20] UNICEF (2003). School Sanitation and Hygiene Education: Scaling Up with Quality.

[21] WHO/UNICEF Joint Monitoring Programme (2004). Meeting the MDG Drinking Water and Sanitation Target: A Mid-Term Assessment of Progress.

[22] Carroll C., Patterson M., Wood S., Booth A., Rick J., Balain S. (2007). A conceptual framework for implementation fidelity. Implement. Sci..

[23] Proctor E., Silmere H., Raghavan R., Hovmand P., Aarons G., Bunger A., Griffey R., Hensley M. (2011). Outcomes for implementation research: Conceptual distinctions, measurement challenges, and research agenda. Adm. Policy Ment. Health.

[24] Hartung C., Lerer A., Anokwa Y., Tseng C., Brunette W., Borriello G. Open Data Kit: Tools to Build Information Services for Developing Regions. Proceedings of the 4th ACM/IEEE International Conference on Information and Communication Technologies and Development.

[25] Adams J., Bartram J., Chartier Y., Sims J. (2009). Water, Sanitation and Hygiene Standards for Schools in Low-Cost Settings.

[26] Vyas S., Kumaranayake L. (2006). Constructing socio-economic status indices: How to use principal components analysis. Health Policy Plan..

[27] Brumback B.A., He Z., Prasad M., Freeman M.C., Rheingans R. (2014). Using structural-nested models to estimate the effect of cluster-level adherence on individual-level outcomes with a three-armed cluster-randomized trial. Stat. Med..

[28] Jordanova T., Cronk R., Obando W., Medina O.Z., Kinoshita R., Bartram J. (2015). Water, sanitation, and hygiene in schools in low socio-economic regions in Nicaragua: A cross-sectional survey. Int. J. Environ. Res. Public Health.

[29] Morgan C., Bowling M., Bartram J., Lyn Kayser G. (2017). Water, sanitation, and hygiene in schools: Status and implications of low coverage in Ethiopia, Kenya, Mozambique, Rwanda, Uganda, and Zambia. Int. J. Hyg. Environ. Health.

[30] Karon A.J., Cronin A.A., Cronk R., Hendrawan R. (2017). Improving water, sanitation, and hygiene in schools in Indonesia: A cross-sectional assessment on sustaining infrastructural and behavioral interventions. Int. J. Hyg. Environ. Health.

[31] Garn J.V., Caruso B.A., Drews-Botsch C.D., Kramer M.R., Brumback B.A., Rheingans R.D., Freeman M.C. (2014). Factors associated with pupil toilet use in kenyan primary schools. Int. J. Environ. Res. Public Health.

[32] Njuguna V., Karanja B., Thuranira M., Shordt K., Snel M., Cairncross S., Biran A., Schmidt W.P. (2008). The Sustainability and Impact of School Sanitation, Water and Hygiene Education in Kenya.

[33] Pickering A.J., Davis J., Blum A.G., Scalmanini J., Oyier B., Okoth G., Breiman R.F., Ram P.K. (2013). Access to waterless hand sanitizer improves student hand hygiene behavior in primary schools in Nairobi, Kenya. Am. J. Trop. Med. Hyg..

[34] Saboori S., Greene L.E., Moe C.L., Freeman M.C., Caruso B.A., Akoko D., Rheingans R.D. (2013). Impact of regular soap provision to primary schools on hand washing and E. coli hand contamination among pupils in Nyanza Province, Kenya: A cluster-randomized trial. Am. J. Trop. Med. Hyg..

[35] Trinies V., Ghulamali S., Freeman M.C. (2015). Dubai Cares WASH in Schools Initiative in Mali Impact Evaluation Report.

[36] Curtis V.A., Danquah L.O., Aunger R.V. (2009). Planned, motivated and habitual hygiene behaviour: An eleven country review. Health Educ. Res..

[37] Hirai M., Graham J.P., Mattson K.D., Kelsey A., Mukherji S., Cronin A.A. (2016). Exploring Determinants of Handwashing with Soap in Indonesia: A Quantitative Analysis. Int. J. Environ. Res. Public Health.

