# Supplementary material for: Design, Intervention Fidelity, and Behavioral Outcomes of a School-Based Water, Sanitation, and Hygiene Cluster-Randomized Trial in Laos
Source: Int J Environ Res Public Health. 2018 Mar 22;15(4):570. doi: 10.3390/ijerph15040570 (PMC5923612; doi:10.3390/ijerph15040570)
Supplement: Supplementary file 1 [file ijerph-15-00570-s001.pdf]

Table S1. Key school-level indicators by intervention status at baseline

	**Intervention** (n=50)	**Comparison** (n=50)	*p*
Mean (SD) total number of enrolled students	72.0 (8.3)	67.8 (7.8)	0.71
Mean (SD) total number of girls enrolled	33.9 (3.6)	32.2 (3.6)	0.74
Mean (SD) total number of boys enrolled	38.1 (4.8)	35.5 (4.4)	0.70
Gender parity (boys/girls)	1.12 (0.04)	1.11 (0.4)	0.49
Pupil absent on day of roll-call[1]	761 (21.2%)	564 (16.7%)	0.72
Presence of latrine at school	9 (18.0%)	11 (22.0%)	0.62
Water point on school compound	7 (29.2%)	4 (16.7%)	0.30
Presence of handwashing facilities at school	0 (0%)	0 (0%)	--
Complete school (vs. incomplete)	21 (42.0%)	15 (30.0%)	0.21
Mean (SD) amount of School Block Grant (in USD)	$320 ($226)	$234 ($158)	0.05

[1]n=3,598 in intervention group; n=3,388 in comparison group

Table S2. Key pupil-level indicators by intervention status at baseline

	**Intervention n(%)** 1,120 (51.5%)	**Comparison n(%)** 1,056 (48.5%)	*p*
Mean (SD) pupil age[1]	10.4 (2.0)	10.5 (1.9)	0.61
Mean (SD) pupil grade	3.8 (0.8)	3.7 (0.8)	0.43
Gender: Girl	547 (48.8%)	520 (49.2%)	0.92
Improved water source used at home	773 (69.1%)	600 (56.8%)	0.78
Toilet at home	198 (17.7%)	269 (25.5%)	0.71
Presence of handwashing facility at home	470 (42.0%)	451 (42.7%)	0.46
Above median household wealth (ref: below)	500 (45.6%)	559 (53.5%)	0.94
Absence in previous week	193 (17.3%)	173 (16.4%)	0.84
Diarrhea in previous week	90 (8.1%)	88 (8.3%)	0.70
Respiratory infection symptoms in previous week	318 (28.4%)	303 (28.7%)	0.58
STH infection[2]	780 (41.2%)	721 (40.9%)	0.95

[1]Age unknown for 218 pupils (Intervention=107, Comparison=111)

[2]n=1,894 in intervention group; n=1,765 in comparison group

Table S3. Beta coefficients (β) and 95% confidence intervals (CI) of the associations between school toilet output criteria and percentage of pupils reported toilet use for last defecation during the school day

	**At least 1 toilet meeting criteria**		**Per toilet meeting criteria**	
**Criteria**	**β**	**95% CI**	**β**	**95% CI**
Toilet output (unlocked, gender separated, water available for flushing, clean)	*20.1*	*(14.0, 26.2)*	*6.4*	*(4.2, 8.6)*
Unlocked	*35.4*	*(28.4, 42.4)*	*12.0*	*(9.7, 14.4)*
Gender separated	7.5	(-1.8, 16.7)	1.3	(-1.8, 4.3)
Water for flushing	*13.0*	*(5.7, 20.3)*	*2.9*	*(0.1, 5.8)*
Clean	*25.6*	*(14.0, 37.1)*	1.2	(-3.1, 5.5)

Bold italics indicate statistical significance at $p<0.05$
